# The extreme capsule fiber complex in humans and macaque monkeys: a comparative diffusion MRI tractography study

**DOI:** 10.1007/s00429-015-1146-0

**Published:** 2015-12-01

**Authors:** Rogier B. Mars, Sean Foxley, Lennart Verhagen, Saad Jbabdi, Jérôme Sallet, MaryAnn P. Noonan, Franz-Xaver Neubert, Jesper L. Andersson, Paula L. Croxson, Robin I. M. Dunbar, Alexandre A. Khrapitchev, Nicola R. Sibson, Karla L. Miller, Matthew F. S. Rushworth

**Affiliations:** 1 Oxford Centre for Functional MRI of the Brain, Nuffield Department of Clinical Neurosciences, John Radcliffe Hospital, University of Oxford, Oxford, OX3 9DU UK; 2 Donders Institute for Brain, Cognition and Behaviour, Radboud University Nijmegen, 6525 EZ Nijmegen, The Netherlands; 3 Department of Experimental Psychology, University of Oxford, South Parks Road, Oxford, OX1 3UD UK; 4 Oxford Centre for Human Brain Activity, Department of Psychiatry, Warneford Hospital, University of Oxford, Oxford, OX3 7JX UK; 5 Icahn School of Medicine at Mount Sinai, New York, NY 10029-6574 USA; 6 Cancer Research UK/Medical Research Council Oxford Institute for Radiation Oncology, Department of Oncology, University of Oxford, Oxford, OX3 7DQ UK

**Keywords:** Tractography, Comparative neuroscience, Temporal cortex, Ventrolateral prefrontal cortex, Surface projection

## Abstract

We compared the course and cortical projections of white matter fibers passing through the extreme capsule in humans and macaques. Previous comparisons of this tract have suggested a uniquely human posterior projection, but these studies have always employed different techniques in the different species. Here we used the same technique, diffusion MRI, in both species to avoid attributing differences in techniques to differences in species. Diffusion MRI-based probabilistic tractography was performed from a seed area in the extreme capsule in both human and macaques. We compared in vivo data of humans and macaques as well as one high-resolution ex vivo macaque dataset. Tractography in the macaque was able to replicate most results known from macaque tracer studies, including selective innervation of frontal cortical areas and targets in the superior temporal cortex. In addition, however, we also observed some tracts that are not commonly reported in macaque tracer studies and that are more reminiscent of results previously only reported in the human. In humans, we show that the ventrolateral prefrontal cortex innervations are broadly similar to those in the macaque. These results suggest that evolutionary changes in the human extreme capsule fiber complex are likely more gradual than punctuated. Further, they demonstrate both the potential and limitations of diffusion MRI tractography.

## Introduction

The macaque monkey is a key model in the study of many aspects of brain organization and function. Despite the rise of in vivo neuroimaging methods there are still many types of information that generally cannot be obtained in humans, such as direct neuronal recordings and the effects of controlled, specific lesions on neural activity and behavior. A major challenge for neuroscience is to establish how well results obtained using one method and in one species generalize to other methods and other species (Passingham 2009).

One area in which the issue of generalizability of results is particularly prominent is in the study of the architecture of connections between brain areas. Connectivity research traditionally relied on studies using neural tract tracers in the monkey model (Morecraft et al. 2009). This technique is laborious and expensive, but yields extremely detailed information about the connections of any injected area. The results of decades of studies using this technique are now combined in databases such as CoCoMac (Bakker et al. 2012) providing a detailed understanding of the connections of the macaque monkey brain, that is being refined continuously (Markov et al. 2014). This approach has recently been complemented by the development of diffusion MRI and related tractography methods that aim to provide an in vivo approach to quantifying the architecture of connections in the human brain (Johansen-Berg and Behrens 2013). Although the results from diffusion MRI tractography studies in humans have yielded results that are generally quite similar to the tracer results obtained in monkeys (Catani et al. 2003; Croxson et al. 2005; Rushworth et al. 2006), some differences have also been identified (Rilling et al. 2008; Thiebaut de Schotten et al. 2012). In most cases, such differences have been attributed to differences between species that have evolved since their last common ancestor.

A particularly compelling case is presented by the complex of white matter fibers that pass through the extreme capsule, a pathway that in the human brain has been identified as crucial for the transfer of information between areas involved in language (Weiller et al. 2011; Friederici and Gierhan 2013). Early non-human primate work describes a fiber pathway located between the claustrum and the insular cortex that forms part of a cortical association bundle interconnecting frontal, insular, and temporal cortices [Berke (1960), cited in Schmahmann and Pandya (2006)]. A series of tracer studies have focused on the extreme capsule fiber complex (ECFC) as the principal fiber bundle connecting the middle part of the superior temporal region with the lateral prefrontal cortex (Petrides and Pandya 1988; Schmahmann and Pandya 2006). Recent studies of the connectivity of the macaque homologue of Broca’s area (Petrides et al. 2005) show that the ECFC connects superior temporal gyrus and sulcus and the dorsal part of inferior temporal cortex with area 45 and, more moderately, with area 44 (Petrides and Pandya 2009; Frey et al. 2014).

In humans, dissection and more recently diffusion MRI studies have identified a fiber bundle through the extreme capsule that connects to similar areas in the frontal cortex, but the projection areas in the posterior part of the cortex have been debated. Frey and colleagues showed that ECFC connects to similar temporal areas in humans as expected from the macaque monkey (Frey et al. 2008), while other authors have emphasized connections to more posterior inferior parietal areas in the human (Makris and Pandya 2009). Catani and colleagues have identified a long horizontal pathway in a similar location in humans, which they termed the inferior fronto-occipital fascicle in reference to terminology used in 19th century anatomical descriptions [see Forkel et al. (2014) for a comprehensive review], that reaches all the way to the visual cortex, part of which they argue may not be present in the macaque and which may therefore be related to our unique behavioral repertoire (Thiebaut de Schotten et al. 2012; Forkel et al. 2014). Thus, both complete or partial overlap between human and macaque ECFC and significant between-species differences, most notably extended posterior projections in the human brain, have been suggested in the literature.

These conclusions are based not just on a comparison between species—macaques and humans—but also on a comparison between methods—tract tracing and diffusion MRI. Here, we evaluate the claims of macaque and human comparative studies by studying the course and projection areas of the ECFC in both species using the same diffusion MRI method. We collected diffusion MRI data from six macaque monkeys in vivo and one ex vivo macaque and compared them to high-resolution data from six human participants obtained in vivo by the Human Connectome Project.

## Materials and methods

We used diffusion-weighted MRI to trace the pathways of fibers passing through the extreme capsule in humans and macaques. All data were preprocessed, analyzed and visualized using tools from the FMRIB Software Library [Smith et al. (2004), http://www.fmrib.ox.ac.uk/fsl], the in-house MR Comparative Anatomy Toolbox (Mr Cat, http://www.neuroecologylab.org) for Matlab (The Mathworks), and the Connectome Workbench [Marcus et al. (2011), http://www.humanconnectome.org]. Individual images were co-registered to account for eddy current and *B*_0_ drift using affine registration using FLIRT (Jenkinson and Smith 2001), if necessary skull stripped using BET (Smith 2002) and hand corrected, and diffusion tensor fitting results were produced using DTIFIT (Behrens et al. 2003). Other preprocessing steps specific to each dataset are described below.

### Human data

Human in vivo diffusion MRI data were provided by the Human Connectome Project (HCP), WU-Minn Consortium (Principal Investigators: David Van Essen and Kamil Ugurbil; 1U54MH091657) funded by the 16 NIH Institutes and Centers that support the NIH Blueprint for Neuroscience Research; and by the McDonnell Center for Systems Neuroscience at Washington University (Van Essen et al. 2013). The minimally preprocessed datasets of the first six subjects (4 female, age range 22–35 years) from the Q2 public data release were used. Data acquisition and preprocessing methods are detailed in Ugurbil et al. (2013), Sotiropoulos et al. (2013) and Glasser et al. (2013). In brief, 1.25 mm isotropic resolution data were collected across the entire brain on a customized 3T Siemens Skyra scanner using a monopolar Stejskal-Tanner diffusion scheme with a slice-accelerated EPI readout. Sampling in *q*-space included 3 shells at *b* = 1000, 2000, and 3000 s/mm^2^. For each shell 90 diffusion gradient directions and 6 *b* = 0′s were acquired with reversed phase-encoding direction that were subsequently combined using the FSL TOPUP distortion correction tool (Andersson et al. 2003). In addition, we used a 32 k surface created using FreeSurfer on the T1-weighted image (acquired using an MPRAGE sequence at 0.7 mm isotropic resolution) and aligned to the diffusion space as part of the HCP’s minimum preprocessing pipeline (Glasser et al. 2013) to display the data.

### Macaque in vivo data

In vivo diffusion MRI data were obtained from six rhesus monkeys (*Macaca mulatta*, 3 female, average age 5.12 years) using a 3T whole-body scanner at the University of Oxford. Protocols for animal care, magnetic resonance imaging, and anesthesia were carried out under authority of personal and project licenses in accordance with the UK Animals (Scientific Procedures) Act (1986) using similar procedures to those that we have described previously (Sallet et al. 2011; Noonan et al. 2014). During scanning animals were kept under minimum anesthetic using isoflurane and kept under continuous veterinary observation. A four-channel phased-array radio-frequency coil in conjunction with a local transmission coil was used for data acquisition (Windmiller Kolster Scientific, Fresno, CA, USA). Diffusion MRI scanning was performed using a twice-refocused diffusion-weighted spin echo sequence (Reese et al. 2003) with TE/TR: 102 ms/8.3 s; resolution: 1 × 1 mm; slice thickness: 1 mm; 60 isotropically distributed diffusion directions; *b* = 1000 s/mm^2^. To correct for image distortion, images were acquired with both left-right and right-left phase encoding direction and subsequently combined using TOPUP, as described above (Andersson et al. 2003). Six datasets were acquired in a single session and averaged to create a single set of data.

### Macaque ex vivo data

Ex vivo diffusion MRI data were obtained from one rhesus monkey (*Macaca mulatta*, male, age at death 4.03 years) using a 7T magnet with a Varian DirectDrive™ (Agilent Technologies, Santa Clara, CA, USA). The brain was perfusion fixed with formalin and subsequently placed in an agar gangue for scanning purposes. Seven non-diffusion-weighted (*b* = 0 s/mm^2^) and 60 diffusion-weighted (*b* = 4000 s/mm^2^) volumes were acquired using a single line readout, 2D Stejskal-Tanner pulse sequence (Stejskal and Tanner 1965) (TE/TR: 25 ms/10 s; matrix size: 128 × 128; resolution 0.6 mm × 0.6 mm; 128 slices; slice thickness: 0.6 mm; 60 isotropically distributed diffusion directions; receiver bandwith: 100 kHz). All co-registered *b* = 0 s/mm^2^ and *b* = 4000 s/mm^2^ datasets were averaged to create a single set data. The diffusivity of ex vivo tissue is generally reduced due to death and fixation, while diffusion anisotropy is largely preserved (D’Arceuil and De Crespigny 2007). This change in diffusivity necessitates the use of larger b-values to achieve equivalent diffusion contrast to in vivo data, as is achieved here by increasing the diffusion coefficient from *b* = 1000 to 4000 s/mm^2^ (Dyrby et al. 2011; Miller et al. 2011).

### Data analysis

Following the preprocessing steps described above, voxel-wise model fitting of diffusion orientations and tractography were performed in a similar way in the human and macaque datasets as follows. We used BedpostX to fit a crossing fiber model to the data (Behrens et al. 2007). For the human in vivo data, a multi-shell extension was used to reduce overfitting of crossing fibers due to non-monoexponential diffusion decay (Jbabdi et al. 2012). Up to three fiber orientations per voxel were allowed. This produced voxel-wise posterior distributions of fiber orientations that were subsequently used in probabilistic tractography.

For both species and for each hemisphere, a mask in the ECFC was drawn in standard space (FSL’s MNI152 brain for the human data, the monkey MNI template of Frey et al. (2011) for the macaque data) in coronal slices at the level of the temporal pole. This mask was subsequently warped to each individual’s diffusion space using FNIRT and hand corrected to include only voxels belonging to the fibers between the insula and putamen. Given the resolution of the data, it is likely that the seed mask contained fibers belonging both to the ECFC and the external capsule, which is separated from the ECFC only by the claustrum but which is considered to be a strictly corticostriatal fiber system (Schmahmann and Pandya 2006). In the interest of brevity we will refer to the seed region only as ECFC. In humans, the center of gravity in MNI space of the ECFC seeds was at [24, 12, −3] for the right and [−26, 11, −4] in the left hemisphere.

To constrain the tractography algorithm, exclusion masks for each individual were drawn through the mid-sagittal slice, through the internal capsule in both hemispheres, through the superior fronto-occipital fascicle as defined by Schmahmann and Pandya (2006) in macaques and Makris et al. (2007) in humans in both hemispheres, and through the fornix. Because it is often difficult to avoid finding fibers belonging to the other main pathway between frontal and temporal cortex, i.e., the dorsal pathway through the arcuate fascicle, we also used an anterior and a posterior coronal exclusion mask that extended from the top of the scan to the bottom of the corpus callosum while avoiding the temporal lobe. Finally, an axial exclusion mask just above the corpus callosum between these two coronal exclusion masks served to constrain tractography in the dorsal–ventral dimension. Any streamlines coming into contact with any of the exclusion masks were discarded from the tractography results.

Five thousand sample streamlines were seeded from each voxel within each individual’s ECFC seed mask. The following parameters were used when running the tractography algorithm for the macaque in vivo, macaque ex vivo, and human in vivo data, respectively: maximum of 2400, 3840, and 3200 steps; step size of 0.25, 0.125, and 0.3125 mm; curvature threshold of 0.2. Each streamline followed local orientations sampled from the posterior distribution given by BedpostX, as described previously (Behrens et al. 2007). A visitation map or tractogram was constructed for each individual. To allow comparison of these maps between subjects and between species the maps were normalized by log transforming the data and dividing each voxel’s value by that of the maximum value in the map yielding voxel values between 0 and 1, and thresholded.

For illustration purposes, we recorded the number of samples that hit the grey/white matter border and projected them on a surface representation of the brain, creating a vertex tractogram. The surface data of each vertex were log transformed and normalized analogously to the volume tractograms. In some cases, we used connectivity fingerprints (Passingham et al. 2002) to illustrate the pattern of connectivity of EmC with predefined target areas. The value reported on the fingerprint represent the average value of the vertex tractograms within area (or ROI) across subjects, and normalized to maximum and minimum values of the selected ROIs. This procedure means that the connectivity fingerprints will serve to illustrate the pattern of EmC connections across the areas in the fingerprint, but that the values on the axes of the fingerprints cannot be compared between fingerprints. Therefore, the original values representing the average tractogram strengths are displayed on the connectivity fingerprint plots.

## Results

### Human in vivo data

**Fig. 1:**
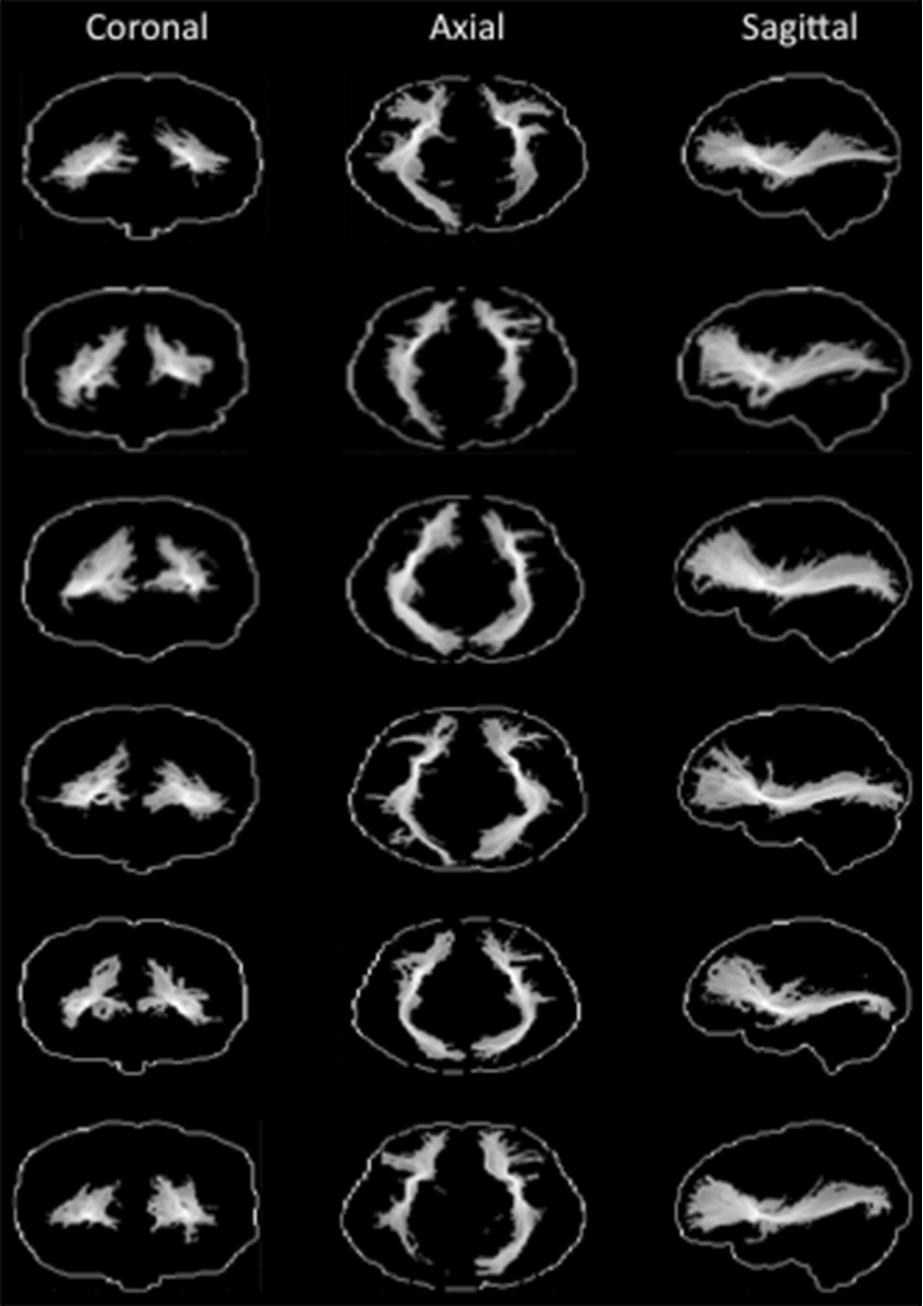
Single subject human tractography results. For each subject the normalized tractograms seeded in left and right ECFC are displayed on glass brains (thresholded at 0.5); for each orientation the maximum intensity voxel across the collapsed dimension is displayed

**Fig. 2:**
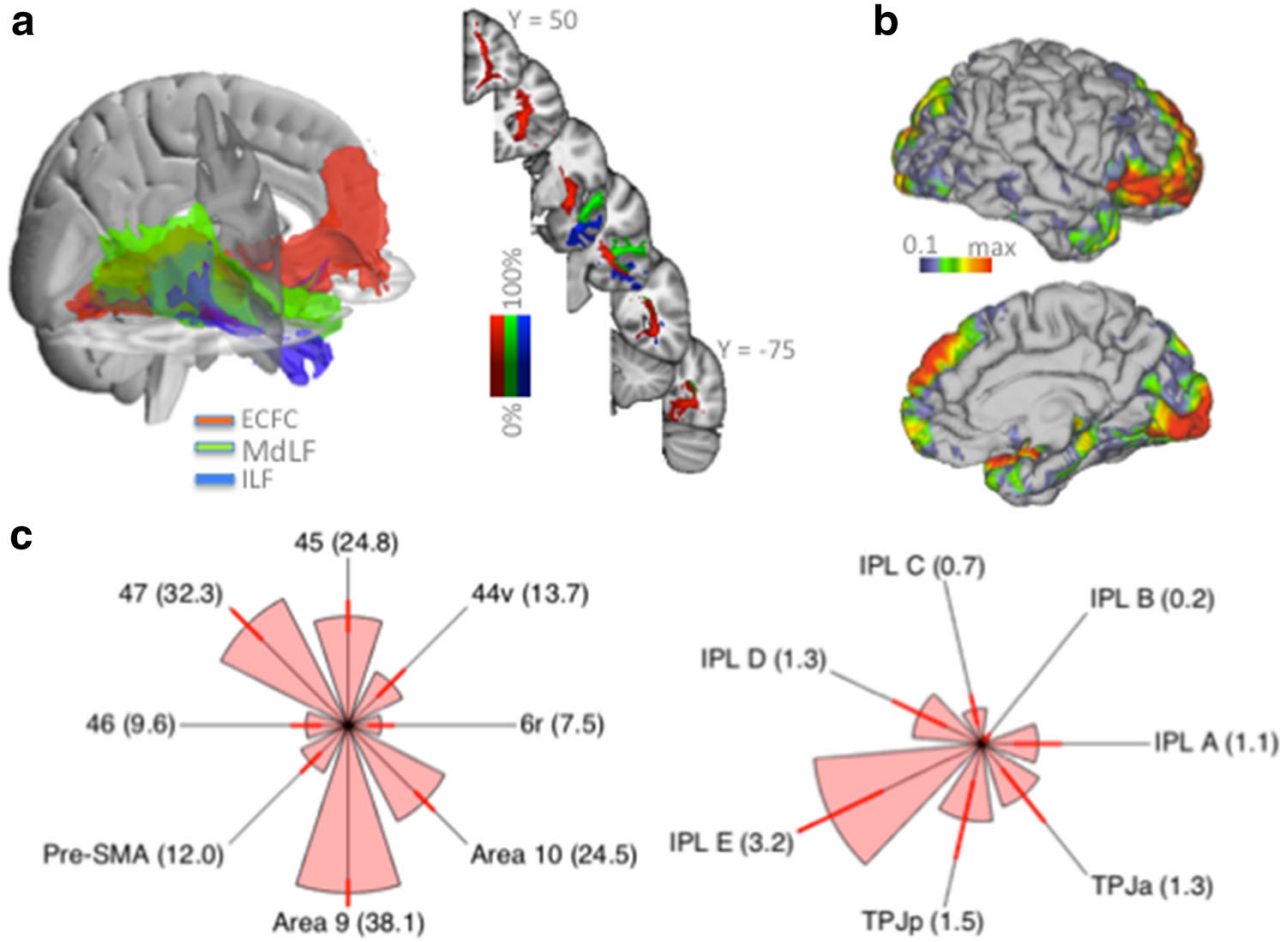
Group human tractography results. **a** Group averaged tractograms (rank threshold of 0.9) of the ECFC (*red*), MdLF (*green*), and ILF (*blue*), and percentage overlap of the single subject results displayed in Fig. 1 (*right panel*). **b** Surface projection of ECFC tractogram. **c** Connectivity fingerprints of the ECFC surface projection to frontal (*left panel*) and inferior parietal and temporoparietal (*right panel*) areas. Arms of the fingerprints represent group average; *error bars* indicate standard errors; fingerprints are scaled to the maximum and minimum connection strength of the target areas; *numbers* in *brackets* indicate average connection strength normalized to 100 being the strongest connection in the brain

The result of each human participant’s tractography seeded in ECFC is shown in Fig. 1. The normalized tractograms, thresholded at 0.5, show a ‘bow tie-shaped’ fiber bundle in the sagittal plane between the frontal and temporal lobes, consistent with previous observations of the course of the ECFC. This tractogram was consistent across subjects and hemispheres. We created a group representation of the course of the ECFC by averaging the individual subjects’ normalized tractograms for the right hemisphere. As can be seen in Fig. 2a (left panel), the ECFC extended as far frontally as the frontal pole and as far posteriorly as the occipital lobe. Both these extremes of the fiber were highly reliable across subjects (Fig. 2a, right panel). Throughout the temporal lobe the ECFC takes a rather medial course, fanning out in the ventral-dorsal dimension.

We performed similar analyses for the other major longitudinal white matter fibers running along the temporal lobe, the middle longitudinal fascicle (MdLF, green in Fig. 2a) and the inferior longitudinal fascicle (ILF, blue in Fig. 2a). The seeds were placed about 15 mm more posteriorly than the ECFC seed in the superior and middle frontal gyri. These seed locations were chosen because (1) the ILF and MdLF do not run through the extreme capsule between claustrum and insula and (2) we wanted to be able to define the facts unambiguously in the superior and middle/inferior temporal gyrus. The seeds were placed in each individual brain, resulting in an average center of gravity of [43, −6, −28] and [43, −5, −6], respectively. As can be seen in Fig. 2a, both tracts remained separated from the ECFC, both coursing more laterally in the gyri of the temporal lobe.

Although the course of a fiber bundle is important, the ultimate goal of most tract tracing studies is to establish which parts of the grey matter are reached, in other words which areas are connected through a given tract. We therefore investigated the number of times the tractogram seeded in the extreme capsule reached a vertex on the grey/white matter border of the cortex to create a projection map of the ECFC. Individual projection maps were normalized and averaged to create a group projection (Fig. 2b). Frontally, ECFC tracts mostly innervated ventral areas around the inferior frontal cortex and the anterior medial frontal cortex. Similar to what was observed in the macaque, these fibers reached the anterior parts of the medial surface in the vicinity of areas 32, 9, and 10. On the more dorsal part of the frontal cortex, the tracts reached the vicinity of dorsolateral prefrontal areas 9 and 46 (Sallet et al. 2013). Posteriorly, the occipital projections of the ECFC are clearly visible, as are projections to the most posterior part of the inferior parietal cortex, at the junction of parietal, temporal, and occipital cortex.

To relate these ECFC projections to anatomical areas, we compared the location of the projections with those of previously published anatomical atlases. These atlases were created based on previous connectivity-based parcellation studies from our group and are freely available online (http://www.neuroecologylab.org). Importantly, the areas in these atlases have all been related to homologs in the macaque, allowing a comparison of the tractogram projections with results obtained in the macaque monkey using different methods. Macaque ECFC reached ventrolateral prefrontal areas, but did not innervate the premotor cortex (Petrides and Pandya 2009). Dorsally, ECFC fibers reached as far as the dorsolateral prefrontal cortex (Petrides and Pandya 1988), while medially ECFC reached prefrontal cortex, but not the more posterior supplementary motor cortex. To test whether this holds for human ECFC, we investigated the projections to areas 6r, 44v, 45, and 47 as defined by Neubert et al. (2014) and area 46, the pre-supplementary motor area (pre-SMA), and areas 9 and 10 as defined by Sallet et al. (2013). The normalized surface-projection of each subject’s ECFC tractogram was overlaid with each of the atlas-based regions of interest and the number of streamlines within each of the frontal subregions (thresholded at >50 % of the population) was calculated and represented as a connectivity fingerprint (Fig. 2c). This showed that, consistent with results obtained from the macaque tract tracing literature, ECFC tended to project preferentially to more anterior-ventral parts of the ventro-lateral prefrontal cortex, reaching mostly areas 47 and 45. Area 44 was reached much less often and ventral premotor areas 6r received hardly any projections. In the medial and dorsal prefontal cortex, strong projections to medial areas 9 and especially 10 were evident, with much weaker projections to pre-SMA and area 46.

The projections to the inferior parietal lobule (IPL) and adjacent temporo-parietal cortex were investigated in an analogous manner, using the five anterior-to-posterior IPL regions (IPL A to IPL E) defined by Mars et al. (2011) and the anterior and posterior parts of the temporoparietal junction area (TPJa and TPJp, respectively) as defined by Mars et al. (2012). As can be seen in Fig. 2c, ECFC reached mostly posterior IPL regions, with the posterior angular gyrus (IPL E) the strongest target, followed by the more ventral TPJp and anterior angular gyrus (IPL D). It should be noted that the strength of the connections as displayed on the axes of the IPL fingerprint should not be compared directly with those of the frontal fingerprint, as the overall connection strength to inferior frontal cortex was much stronger than to IPL, as indicated by the number representing the average normalized projection strength of the fingerprints in Fig. 2c.

### Macaque in vivo data

The results obtained in the human generally showed results similar to what would be predicted based on the macaque tracer literature. The main exception was formed by the very posterior projections of the ECFC that, although compatible with the results of previous human diffusion MRI tractography studies, was not immediately compatible with the macaque tracer literature. To investigate whether this is a genuine between species difference or whether this is due to the fact that macaque studies generally do not use the same method, we next investigated tractography from seeds in the extreme capsule in six in vivo macaque diffusion MRI datasets.

**Fig. 3:**
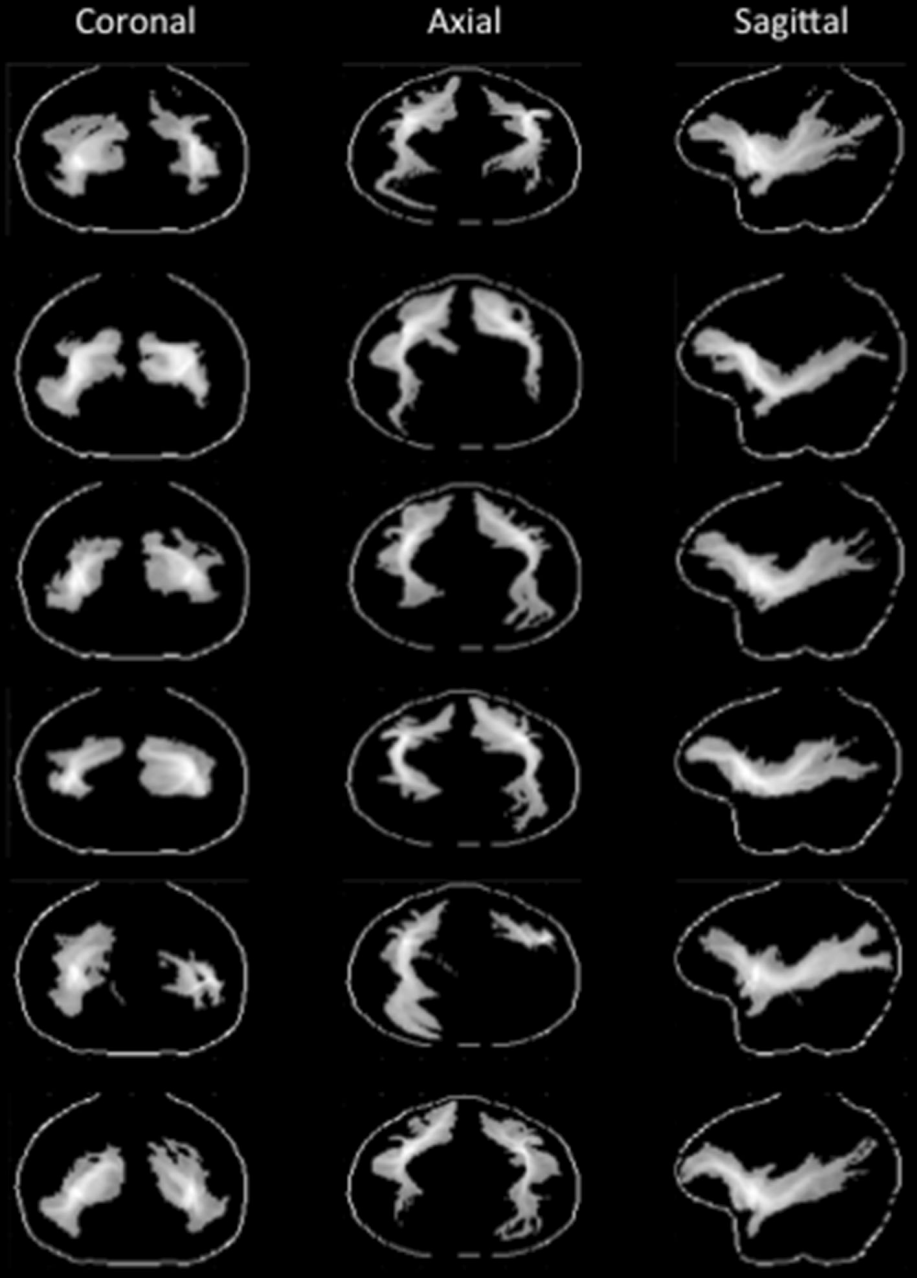
Single subject macaque tractography results. For each subject the normalized tractograms seeded in left and right ECFC are displayed on glass brains (thresholded at 0.5); for each orientation the maximum intensity voxel across the collapsed dimension is displayed

The result of each macaque’s tractography seeded in ECFC is shown in Fig. 3. The group tractogram confirmed some of the broad results obtained in macaque tracer studies. MRI-defined ECFC tracts cruised near ventrolateral prefrontal areas and reached the territory around the principal sulcus that contains area 9/46. The tractogram also reached a more medial area anterior to the genu of the corpus callosum containing area 32 and, more anteriorly, areas 9 and 10, all of which are known to be reached by ECFC fibers (Petrides and Pandya 1988; Schmahmann and Pandya 2006; Petrides and Pandya 2007).

Posteriorly, the tractogram of all monkeys ran adjacent to the superior temporal sulcus in an anterior-posterior direction. The middle part of the superior temporal sulcus is known to contain major targets of ECFC fibers in the macaque (Petrides and Pandya 1988; Seltzer and Pandya 1989; Schmahmann and Pandya 2006; Frey et al. 2014) and the tractography data showed that evidence for similar connections was replicated in all macaque subjects. In most hemispheres, the tractogram ran further posteriorly, which is not directly compatible with the proposed route of ECFC fibers in macaque (Schmahmann and Pandya 2006), but does resemble the ECFC route proposed for the human on the basis of tractography investigations (Thiebaut de Schotten et al. 2012) and shown in the human data described above. However, as can be seen by comparing Figs. 1 and 3, this result was not quite as consistent as that obtained in the humans. This might be due to the fact that the size of the voxels relative to the size of the brain is actually much coarser in the macaque dataset (1 mm resolution) as compared to the human data. Since we followed the ECFC from a starting point placed quite lateral in the extreme capsule and which had to curve slightly in the lateral-medial dimension, this might have affected the tractography algorithm’s ability to pick up longer fibers. Therefore, we sought to replicate and further investigate these results in a post-mortem macaque brain, which allowed us to acquire data at a more appropriate spatial resolution.

### Macaque ex vivo data

**Fig. 4:**
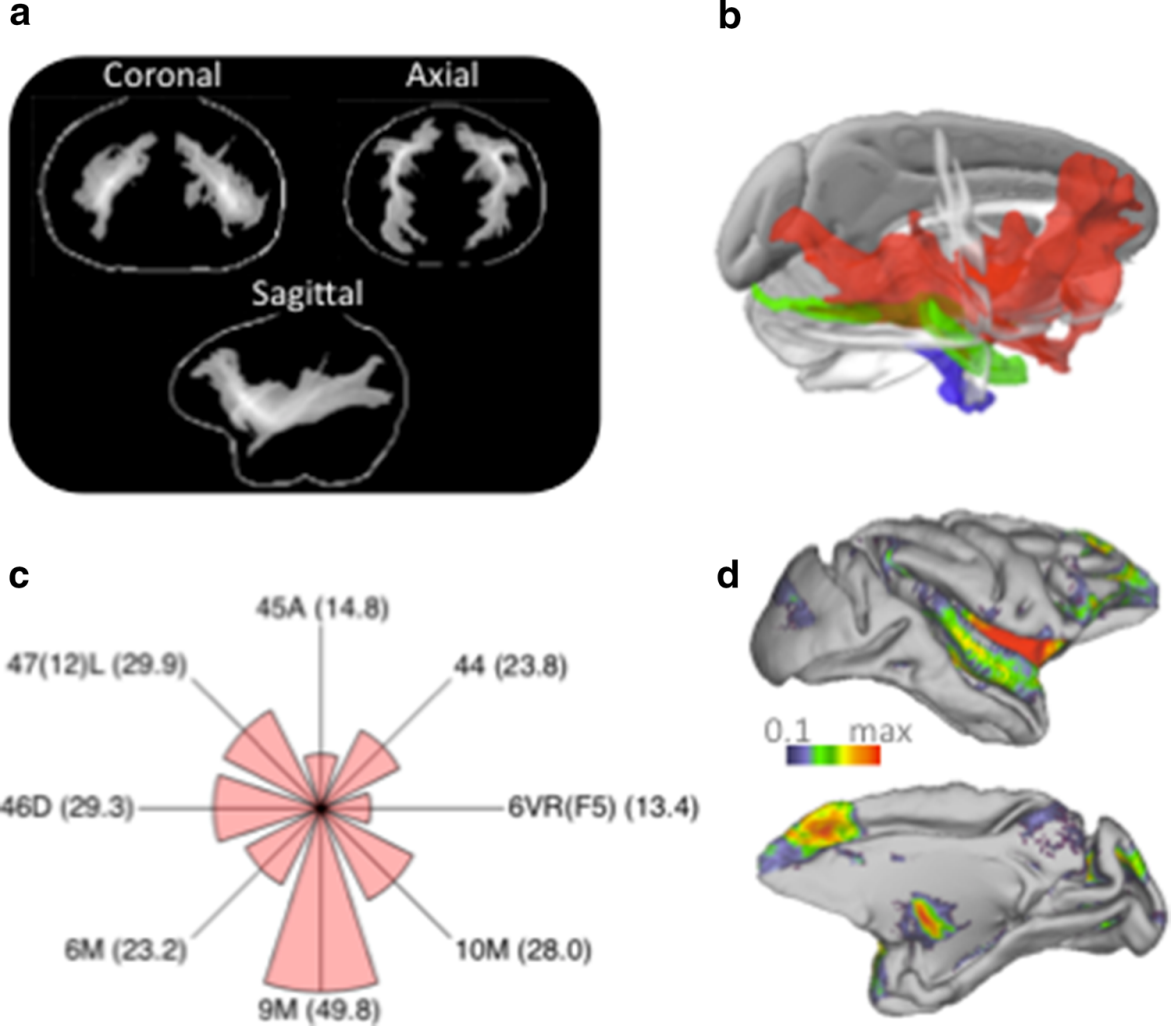
Macaque exvivo tractography results. **a** Normalized tractograms seeded in left and right ECFC are displayed on a glass brain (thresholded at 0.5); for each orientation the maximum intensity voxel across the collapsed dimension is displayed. **b** Tractograms (rank thresholded at 0.7) of the ECFC (*red*), MdLF (*green*), and ILF (*blue*). **c** Connectivity fingerprints of the ECFC surface projection with frontal areas described in the atlas of (Paxinos et al. 2000). Fingerprints are scaled to the maximum and minimum connection strength of the target areas; *numbers* in *brackets* indicate average connection strength normalized to 100 being the strongest connection in the brain. **d** Surface projection of ECFC tractogram displayed on the F99 template brain (Van Essen 2002)

Tractography was run from a seed in the ECFC at the level of the temporal pole in both hemispheres. The resulting tracts were similar in both hemispheres and ran from the ventral prefrontal cortex all the way through the length of the temporal cortex as far back as the visual cortex and posterior part of the inferior parietal lobule around area PG (Fig. 4a). These results again mimiced the observation in humans of a tract seeded in the ECFC reaching much more posteriorly than predicted based on tracer data (Makris and Pandya 2009; Thiebaut de Schotten et al. 2012).

Similar to the analyses run in humans, we also seeded the MdLF and ILF to exclude the possibility that the posterior ECFC projections are originating from these tracts. In separate analyses we therefore explored tractography from seed regions placed in the MdLF and the ILF approximately 1.3 cm posterior to the tip of the temporal pole. Tractograms of the MdLF and ILF in both hemispheres followed the length of the temporal cortex, with the MdLF tracts located more dorsally than the ILF tracts, consistent with their known pathways (Schmahmann and Pandya 2006; Schmahmann et al. 2007). As in the human, both the MdLF and ILF tracts took a different course along the temporal cortex than the ECFC tract (Fig. 4b).

As described above, the human frontal ECFC projections were quite similar to what would be predicted based on the macaque tract tracing literature. To directly relate diffusion MRI tractography to these results, we performed a similar atlas-based analysis as for the human data. Since we only had one ex vivo brain available we averaged across the two hemispheres to increase the signal-to-noise ratio. We transformed the macaque ex vivo tractogram to F99 standard space (Van Essen 2002) and again created a surface projection of the ECFC (Fig. 4d). The projections were then related to areas described in the atlas of Paxinos et al. (2000). Figure 4c shows the connectivity fingerprint of ECFC with areas 6VR(F5) 44, 45A, 47/12(L), 46D, 6, 9, and 10. As in the human, ventrolateral prefrontal projections were mostly to more anterior area 47 and to a lesser extent to areas 44 and 6VR. One major difference between species was in the strength of the projections to area 45. Surprisingly, these projections were very strong in the human but less so in the macaque, even though the tracer data suggests that extreme capsule fibers innervate this area much more than area 44 (Petrides and Pandya 2009). This might be due to the fact that the macaque area 45 for a large part occupies the crown of a gyrus, from which it is sometime difficult to track using diffusion MRI due to the presence of differentially oriented superficial white matter fibers (Reveley et al. 2015). This, however, is at present a speculative post hoc interpretation. On the medial surface the results are similar between species, with area 9 reached more than area 10 and only weak projections reaching 6M.

## Discussion

In this study we set out to compare the course and cortical projections of white matter fibers passing through the extreme capsule in humans and macaques. Previous comparisons of this tract have suggested a uniquely human posterior projection, but these studies have always used different techniques in different species. Here we used the same technique, diffusion MRI, in both species to avoid attributing differences in techniques to differences in species. Diffusion MRI-based probabilistic tractography was performed from a seed area in the ECFC in in vivo human scans and in in vivo and high-resolution ex vivo macaque scans. In humans, we replicated and extended results reported before (Makris and Pandya 2009; Thiebaut de Schotten et al. 2012; Forkel et al. 2014), showing that the ventrolateral prefrontal cortex innervations are broadly similar to those in the macaque but that temporal cortex tracts run very posterior reaching to the visual cortex. Tractography in the macaque was able to replicate the main effects known from macaque tracer studies, including selective innervation of frontal cortical areas and targets in the superior temporal cortex. In addition, however, we also showed some tracts that are not commonly reported in macaque tracer studies: tracts that extended far posteriorly near to the visual cortex. We were able to rule out the possibility that these effects were due to the tractography algorithm spuriously picking up tracts belonging to the middle and inferior longitudinal fascicles. Below, we will discuss these results and what they suggest for the use of diffusion MRI in comparative neuroscience.

### Frontal projections of ECFC in human and macaque

We showed that the projections of human ECFC to the frontal cortex are much as expected from the macaque fiber tracing studies and tractography data, displaying strong ventral prefrontal connectivity, as well as connections to medial areas 9 and 10, and to the dorsolateral prefrontal cortex. Most macaque tracer studies identify the projections of a given tract by performing cytoarchitectonic analyses of the labeled areas. In the absence of such analyses for most human cortical areas, we have related the frontal cortex projections of ECFC to previous atlases based on connectivity-based parcellations of the human cortex (Sallet et al. 2013; Neubert et al. 2014). Such parcellations often identify areas that are very similar to those identified using cytoarchitecture (Caspers et al. 2011; Mars et al. 2011) and allow one to make a more fine-grained determination of projection areas than when relying only on macro-anatomical boundaries (Croxson et al. 2005). The resulting connectivity fingerprint shows that ECFC preferentially innervates prefrontal cortex, not premotor cortex. Within the ventrolateral prefrontal cortex there is an interesting dissociation between area 45, which in the human receives heavy ECFC projections, and area 44, which in these data seems to receive very few. This result mimics macaque tracer data showing that area 45 preferentially receives ECFC projections (Petrides and Pandya 2009). Surprisingly, this result was not obtained in the macaque, where area 45 received relatively few projections, in contrast with the tracing literature (Petrides and Pandya 2009; Frey et al. 2014). We have suggested this might be due to the location of area 45 on the crown of the gyrus, but at present this is a post hoc interpretation. Intriguingly, the role of human area 45 in semantic language processing (Vigneau et al. 2006) might be compatible with an alternative interpretation of increased area 45 connectivity in the human brain. Either way, this result illustrates the caution that is needed when interpreting diffusion MRI tractography data.

### Posterior projections of ECFC in human and macaque

Our macaque tractography results show a more extensive posterior distribution of temporal cortex ECFC fibers in the macaque than expected based on tract tracing studies. In fact, the macaque results are much more similar to human results than previously suggested. One possible explanation for this observation is that, in contrast to tracer studies that often are sensitive only to mono-synaptic connections, tractography does not respect such boundaries. It might therefore be the case that ECFC tractography identifies connections between frontal and superior temporal cortex and, in addition, connections between superior temporal areas and more posterior areas including some in visual cortex. Together, these fibers can then form the ‘extreme capsule fiber complex’ also known as the ‘inferior fronto-occipital pathway’ identified using diffusion MRI. Consistent with this suggestion, recent tracer studies have identified connections from visual areas to temporal areas known to be the target of ECFC projections from the frontal cortex (Borra et al. 2011; Markov et al. 2014). Consistent with the suggestion that the reported ECFC fibers reflect direct connections, recent tracer studies have reported weak direct mono-synaptic connections from frontal to visual areas (Gerbella et al. 2010; Markov et al. 2014). We also reported some, albeit weaker, connections to the inferior parietal lobule in the human and in the macaque. In humans these connections are most evident in the posterior angular gyrus (Mars et al. 2011) and in the macaque in area PG.

The tractography results in macaques lead us to dispute the suggestion that the posterior part of the long tract through the extreme capsule is an evolutionary completely novel innovation in the human brain. In contrast, it seems to be an elaboration of a series of connections that can also be identified in the macaque brain, although it is clearly the case that fibers reaching the most posterior parts of the cortex do seem to be more prominent and much more consistent in the human than in the macaque. We do not dispute recent suggestions that this tract is important for the transfer of information related to language in humans. On the contrary, human ECFC has been implicated in connecting inferior frontal cortex with higher-order auditory areas (Makris and Pandya 2009) and recent tracer data shows that similar areas are connected by this tract in the macaque brain (Petrides and Pandya 2009; Frey et al. 2014). The ECFC has been identified as a major pathway in brain networks for language and social behavior (Friederici and Gierhan 2013; Catani and Bambini 2014, Noonan et al. submitted) and the current results provide some clues that constrain the origin of these pathways in the common ancestor of humans and macaques. A long-range pathway between visual, temporal, and frontal cortex may be utilized for processes as diverse as reading (Yeatman et al. 2013) and semantic and basic syntactic processes (Rolheiser et al. 2011).

We have recently suggested that the middle part of the macaque superior temporal sulcus (STS) might share some anatomical features with the human posterior TPJ (Mars et al. 2013). This human area is commonly active in studies in which participants are asked to attribute belief states to others, so-called mentalizing (Saxe 2006). Mentalizing is thought to be particularly well developed in humans and the suggestion of an anatomical homolog of a region involved in this behavior has important consequences for our understanding of the evolution of human social abilities. The suggestion that human TPJp and macaque mid-STS share certain features was based on a comparison of the functional connectivity profiles of the two areas using resting state fMRI data. If this suggestion is true, it would be expected that TPJp receives input from the major fibers that reach macaque mid-STS. The results obtained here show that this is indeed the case, with TPJp being reached by the ECFC, albeit weakly. This result is also interesting in the light of recent work showing that the integrity of ECFC correlates with the size of people’s social network (Noonan et al. submitted).

Together with the suggestion that the ECFC in the human brain is a major pathway for visual information (Forkel et al. 2014), these results could introduce a more refined hypothesis to be tested: that identifiable sub-components of the extreme capsule fiber complex might have differently adapted over evolution and show independent variability over individuals, similar to recent hypotheses concerning the arcuate fiber complex (Catani et al. 2005).

### Limitations and future directions

Based on the considerations above, we would argue that any comparison between species should be at least complemented by a test of the comparative hypothesis using the same method in the different species (cf. Jbabdi et al. 2013; Catani 2007). The current study provides an illustration of this approach, but substantial improvements can be made.

First, in line with many previous studies, the current study simply placed a seed in a region of interest based on a priori knowledge of human and macaque anatomy. Although this method has been applied successfully in numerous studies, it involves some judgment calls by the experimenter. As discussed above, recent studies on connectivity often exploit a parcellation approach in which a large region of interest is subdivided based on differential long-range connections with the entire brain (Johansen-Berg et al. 2004; Mars et al. 2012; Sallet et al. 2013). The resulting divisions are then used for further analysis. Such a data-driven technique for determining seed areas can potentially be applied to the cortical white matter itself and would yield much more accurate characterization of the locations of distinct tracts in individuals.

Second, the resolution (spatial, angular, and number of shells) in the three data sets we used here was comparable but not completely equivalent. The fact that we see broadly equivalent results with both methods (in vivo and ex vivo) in the monkey with different parameters and in human in vivo and monkey ex vivo data that are of equivalent resolution when brain size is taken into consideration suggests that the most important thing is the overall method used (probabilistic diffusion MRI with multiple fiber directions) and not the exact parameters used. Furthermore, we have attempted in the analysis of our data to ensure the reported measures are comparable between species. This is why the data were normalized in the way they were.

Finally, the use of MRI to characterize whole brain white matter connectivity allows one to study a large range of species at relatively low costs (Mars et al. 2014). Brain architecture can arguably be much better understood if the comparative paradigm is extended to include more species, allowing one to exploit the variance between species, rather than treating it as a nuisance (Preuss 2000). The successful use of post-mortem samples in diffusion MRI studies in this and other studies (D’Arceuil et al. 2007; Miller et al. 2011) makes this approach ever more feasible.

## Conclusion

In sum, a direct comparison of macaque and human extreme capsule projections visualized using the same technique revealed surprising similarities across species, despite earlier suggestions of a uniquely human posterior part of extreme capsule projections. In fact, the current results support a more refined and incremental account of the evolutionary adaptations of the ECFC in the primate lineage. This study illustrates the need to use the same method when comparing the organization of brain connections between species. In addition, although the results of macaque tractography were broadly consistent with results obtained from tracer studies, some differences illustrate the need for caution in interpreting tractography data.
